# Practical Healthcare Epidemiology, 4th Edition

**DOI:** 10.3201/eid2511.190464

**Published:** 2019-11

**Authors:** Edward Lifshitz

**Affiliations:** New Jersey Department of Health, Trenton, New Jersey, USA

**Keywords:** Epidemiologists, epidemiology, public health, infection control, hospitals, healthcare

On September 28, 2014, a 42-year-old Liberian man was admitted to a Texas hospital and quickly placed in isolation with a presumptive diagnosis of Ebola virus disease (EVD), a diagnosis that was subsequently confirmed. When caring for him, staff were advised to use personal protective equipment as recommended by the Centers for Disease Control and Prevention. Unfortunately, the patient died on October 8, and 2 nurses who had cared for him contracted EVD (*1*). This failure of infection control contributed to the public spread of panic, served as a wake-up call to the public health community, and helped convince the Centers for Disease Control and Prevention that EVD should be treated only in specialized hospitals with highly trained personnel (*2*).

The following spring, a different deadly virus spread rapidly through a modern healthcare system. In South Korea, over the course of 2 months, an outbreak of Middle East respiratory syndrome led to 186 confirmed cases and 38 deaths; most of the patients contracted the virus within healthcare facilities (*3*).

These examples may be dramatic, but healthcare-associated infections are acquired daily and affect an estimated 4% of all hospitalized patients (*4*). Patients are infected by microbes that are introduced by our actions (e.g., catheter-associated urinary tract infections), are made more deadly for patients with a frail medical status (e.g., influenza), or are emerging and resistant (e.g., *Candida auris,* carbapenem-resistant *Enterobacteriaceae*).

Although we may never win the war against disease-causing microorganisms, we can celebrate victories. Since the practice of infection prevention and control became a specialty in the 1960s, healthcare-associated infections have decreased substantially (*5*). New leaders in the field of infection prevention and control will always be needed. It is for these emerging experts that Lautenbach and colleagues have updated their book, Practical Healthcare Epidemiology, to a fourth edition (Figure). They set forth to provide “a pragmatic, easy-to-use reference” that provides “real-world advice” as if they were speaking to a person who would be running an infection prevention program and who was just starting in this field. They have succeeded.

**Figure:**
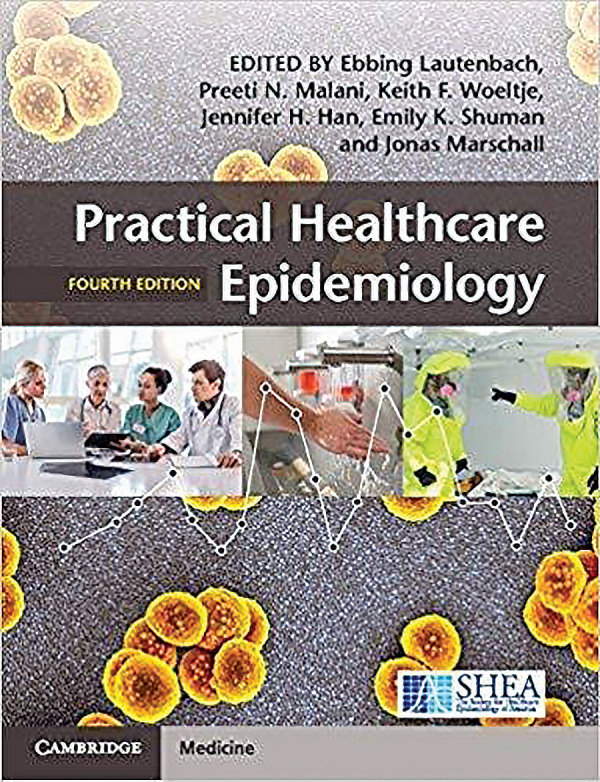
Practical Healthcare Epidemiology, 4th Edition

Practical Healthcare Epidemiology covers a broad range of topics, organized into appropriately themed sections. The authors speak with authority, and there are no weak chapters. The chapter “Ethical Aspects of Infection Prevention,” a subject that is often given short shrift, is itself worth the price of admission.

The 56 listed contributors are a major source of strength, but they also contribute to the book’s minor weaknesses. Organizational consistency is lacking across chapters and even within chapters (e.g., the terms “antibiotic” and “antimicrobial” are used interchangeably). I found it slightly annoying that despite being aimed at “fledgling healthcare epidemiologists,” the book contains no job description for this position and uses “healthcare epidemiologist” and “hospital epidemiologist” synonymously, without explanation.

But enough nit-picking. Practical Healthcare Epidemiology is valuable not just for its intended audience of new practitioners but for anyone involved in infection prevention and control. I especially recommend it for those looking for a practical, easy-to-understand—but not simple—resource.
